# Interaction between Porcine Alveolar Macrophage-Tang Cells and *Streptococcus suis* Strains of Different Virulence: Phagocytosis and Apoptosis

**DOI:** 10.3390/microorganisms11010160

**Published:** 2023-01-08

**Authors:** Siqi Li, Chunsheng Wang, Yan-Dong Tang, Lei Qin, Tianfeng Chen, Shanghui Wang, Yuanzhe Bai, Xuehui Cai, Shujie Wang

**Affiliations:** 1 National Key Laboratory of Veterinary Biotechnology, Harbin Veterinary Research Institute, Chinese Academy of Agricultural Sciences, Harbin 150001, China; 2 College of Life Science, Northeast Forestry University, Harbin 150040, China; 3 Heilongjiang Provincial Key Laboratory of Veterinary Immunology, Harbin 150069, China

**Keywords:** PAM-Tang cell line, *Streptococcus suis*, phagocytosis, apoptosis

## Abstract

*Streptococcus suis* is an important swine bacterial pathogen that activates macrophages to secrete inflammatory cytokines. Primary porcine alveolar macrophages (PAMs) are inconvenient to obtain, but it is unknown whether immortalized PAM-Tang cells can replace them as a better cell model for the study of the interaction between *S. suis* and macrophages. In this study, the phagocytic integrity, polarization, and pro-inflammatory cytokine secretion of PAM-Tang cells were confirmed by live-cell imaging, electron microscopy, confocal microscopy, and ELISA. Interestingly, the *S. suis* serotype 9 avirulent strain W7119 induced higher levels of adhesion and pro-inflammatory cytokines in PAM-Tang cells than the *S. suis* serotype 2 virulent strain 700794. Prolonged incubation with *S. suis* caused more cytotoxic cell damage, and the virulent strain induced higher levels of cytotoxicity to PAM-Tang cells. The virulent strain also induced higher levels of apoptosis in PAM-Tang cells, as shown by terminal deoxynucleotidyl transferase (TdT)-mediated dUTP-biotin nick end labeling (TUNEL) assay. In addition, it is the first report of virulent and avirulent *S. suis* inducing PAM-Tang polarization towards pro-inflammatory M1 macrophages and p53- and caspase-dependent apoptosis in PAMs. Taken together, this study contributes to a better understand of interactions between macrophages and *S. suis* isolates of different virulence, and confirms that PAM-Tang cells provide a long-term, renewable resource for investigating macrophage infections with bacteria.

## 1. Introduction

*Streptococcus suis* is a commensal of the upper respiratory tract of pigs that can cause type-dependent diseases [1] including pneumonia, arthritis, and meningitis [2], which result in great economic losses to the swine industry [3,4]. Due to the frequency of *S. suis* infection in pig herds [5] and the emergence of clinical drug-resistant strains [6,7], in addition to causing immunosuppression [8], it is critically important to understand the interaction between *S. suis* and the immune system.

Macrophages are an essential component of the innate immune system [9], and they show considerable heterogeneity in different microenvironments [10]. They can be classified into two major phenotypes: pro-inflammatory M1 and anti-inflammatory M2 macrophages. As primary immune cells, porcine alveolar macrophages (PAMs) form the first line of defense against *S. suis* infections in the lung, though the impact of strain-specific virulence is unclear. In vitro, *S. suis* serotype 2 shows adhesion and cytotoxicity to primary PAMs [11], murine macrophage cell line J774 and PAM cell line 3D4 [12,13], and induces pro-inflammatory cytokine expression in primary PAMs and PAM cell lines [14], inhibiting activation of signaling pathways involved in phagocytosis after infection of J774 [15].

PAMs are inconvenient to obtain, and long-term storage at −80 °C is unreliable. PAM-Tang cells (including low CD163 abundance and high CD163 abundance) are the immortalization of primary PAMs, developed by introducing SV40 large T antigen through lentiviral transfer plasmid pSFG, which can be cultured with common medium without needing to add special ingredients, and their high CD163 abundance facilitates PRRSV infection, but low CD163 abundances (approximately 20%) do not initiate PRRSV infection [16]. However, it is unknown whether PAM-Tang cells can replace the primary PAMs as a better cell model to study the interaction between *S. suis* and macrophages.

*S. suis* is classified into 29 serotypes, based on the capsular polysaccharide (CPS) antigenicity. Serotype 2 is described as the most virulent serotype [17]. For *S. suis* serotype 2 virulent strain 700794 (vir-700794), the virulence phenotype is *gdh*^+^/*mrp*^+^/*sly*^+^/*epf*^+^, which causes death and severe thymic atrophy in mice [18]. While for *S. suis* serotype 9 avirulent strain W7119 (avir-W7119), the virulence phenotype is *gdh*^+^/*mrp*^−^/*sly*^−^/*epf*^−^, which does does not cause death and any thymic atrophy in mice [18]. Thus, these two strains were selected as representative strains of different virulence in this study.

Even though immortalized macrophage cell lines have been reported as cell models to study the interaction of *S. suis* with macrophages, the mouse-derived cell line J774 is still controversial, and the existing PAM cell line 3D4 requires a special culture medium. The objective of this study was to evaluate the capacity of PAM-Tang cells for phagocytosis and interaction with *S. suis* strains of different virulence (vir-700794 and avir-W7119). In general, PAM-Tang cells were found to be a suitable cell model for the in vitro study of interactions with *S. suis*.

## 2. Materials and Methods

### 2.1. Bacterial Strains and Growth Conditions

*S. suis* serotype 2 virulent strain 700794 (vir-700794) and *S. suis* serotype 9 avirulent strain W7119 (avir-W7119) were used in this study. Both of these strains have previously been used in studies with thymic atrophy [8]. *S. suis* were grown for 20 h at 37 °C on sheep blood agar plates (Thermo, Beijing, China), then a single colony was inoculated into Todd Hewitt broth (THB) (BD, Franklin Lakes, NJ, USA). Bacteria were incubated with agitation until reaching optical density at 600 nm (OD_600_) of 0.6, then diluted appropriately in cell culture medium for infection. The number of CFU/mL in the final suspension was determined by plating samples on Todd Hewitt agar (THA).

### 2.2. Cell Culture

The PAM-Tang cell line was reconstructed by our laboratory, and low CD163 abundance PAM-Tang cells were used in this study, cultured as described previously [16]. Primary PAMs were stored in our lab and also used in this study. Briefly, cells were maintained in RPMI-1640 supplemented with 10% fetal bovine serum (FBS), 100 U/mL penicillin, and 0.1 mg/mL streptomycin at 37 °C in a 5% CO_2_ incubator. For experiments, cell cultures were plated at a density of 1 × 10^6^/well in 12-well culture plates (NEST, Wuxi, China).

### 2.3. PAM-Tang Adhesion Assays

The adhesion assay was performed as previously described [19,20], with some modifications. Briefly, cells were separately infected with virulent or avirulent *S. suis* with a multiplicity of infection (MOI) = 1, by replacing the cell culture media with 1 mL (5 × 10^5^ CFU) of bacterial suspension. After 2, 4, or 8 h of incubation with *S. suis*, cells were washed 4 times to eliminate nonspecific bacterial attachment, adding 0.1% saponin for lysis on ice for 15 min. Then, the lysate was taken and coated on the plate, and the colony count was performed the next day. The levels of adhesion (total associated bacteria) were expressed as the total number of CFU recovered per well.

### 2.4. Cytotoxicity Assay

PAM-Tang cells were infected (MOI = 1) with virulent or avirulent *S. suis* for 0.5, 1, 2, 3, 8, 12, or 24 h, after which culture supernatants were collected and centrifuged for lactate dehydrogenase (LDH) enzyme detection using the CytoTox 96 Non-Radioactive Cytotoxicity assay kit (Promega, Madison, WI, USA). Non-infected cell culture supernatants served as negative controls, and the absorbance of each well was measured at 495 nm wavelength to determine the LDH activity.

### 2.5. Transmission Electron Microscopy

PAM-Tang cells were grown in a 6-well cell culture plate, infected with the *S. suis* 700794 or *S. suis* W7119 (MOI = 1) for 8 h, and the cells were fixed with 4% glutaraldehyde, and further processed for electron microscopy as described previously [21]. The samples were examined on a transmission electron microscope (TEM) HITACHI H-7650 (Tokyo, Japan). The mean number of lysosomes per 20 cells was determined by counting in 10 fields under TEM. The number of pathogens per cell was determined by 10 cells with pathogens in 10 fields under TEM.

### 2.6. Confocal Microscopy

PAM-Tang cells were cultured in glass-bottom cell culture dishes (Biosharp, Hefei, China) and used for double-immunofluorescence staining. To determine the type of cellular polarization after bacterial infection, cell samples were infected with *S. suis* 700794 or *S. suis* W7119 (MOI = 1) for 8 h at 37 °C. The cells were fixed in 4% paraformaldehyde for 30 min, then washed three times with PBS, permeabilized with 0.2% Triton X-100 for 30 min, washed three times again, and blocked with 1% bovine serum albumin (BSA) for 1 h. After blocking, the cells were incubated with rabbit anti-mouse CD206 (1:100, ABclonal, Beijing, China) or rabbit anti-mouse IL-1β (1:200, ABclonal) and mouse anti-*S. suis* serotype 2 or 9 (1:200, made in lab) antibodies for 1 h at room temperature, and then with Alexa Fluor 488-conjugated goat anti-mouse IgG (Invitrogen, Carlsbad, CA, USA) or Alexa Fluor 568-conjugated goat anti-rabbit IgG (Invitrogen, Carlsbad, CA, USA). Apoptosis was detected using terminal deoxynucleotidyl transferase (TdT)-mediated deoxyuridine triphosphate (dUTP)-biotin nick end-labeling (TUNEL) assay, according to the manufacturer’s instructions (In Situ Cell Death Detection Kit; Roche, Mannheim, Germany). Finally, nuclei were stained with 4-6-diamidino-2-phenylindole (DAPI; Sigma). Cell samples were observed on a laser-scanning confocal microscope (Carl Zeiss AG, Oberkochen, Germany).

### 2.7. Cytokine Analysis

To quantify cytokine levels induced by *S. suis* in PAM-Tang cells, all the supernatants were removed from the wells and collected on sequential samples of cell cultures, then replaced with fresh medium at 3, 8, 16, and 24 h post-infection (hpi). The levels of porcine IL-18, IL-1β and TNF-α were detected using commercial enzyme-linked immunosorbent assay (ELISA) kits (XIpcc, Shanghai, China; Cusabio, Hangzhou, China) according to the manufacturer’s instructions. The amount of cytokines (ng/L; pg/mL) was calculated according to a standard curve generated from recombinant controls supplied in the kits.

Infected cells at 3, 8, 16, and 24 hpi were collected and total RNA was extracted by RNA Pre-Pure kit (Tiangen, Beijing, China) according to the manufacturer’s instructions. Then, total RNA was retro-transcribed into complementary DNA using PrimeScript RT reagent Kit (TaKaRa, Dalian, China). SYBR^®^ Green (Vazyme Biotech, Nanjing, China) was used to quantify the PCR-amplification products and GADPH was used as a reference for quantitative RT-PCR (qPCR). Primers were used as follows: IL-6-F: 5′-TGGCTACTGCCTTCCCTACC, IL-6-R: 5′-CAGAGATTTTGCCGAGGATG; IL-8-F: 5′-GCTCCCAAGAATTTCTCAGTA, IL-8-R: 5′-CAGCAGCCTAGGGTTGCAAG; GADPH-F: 5′-TCGGAGTGAACGGATTTGGC, GADPH-R 5′-TGCCGTGGGTGGAATCATAC.

### 2.8. Live-Cell Imaging

PAM-Tang cells were plated in laser confocal dishes in RPMI 1640 medium supplemented with 10% FBS overnight, then the medium was replaced with RPMI 1640 with 3% FBS. To observe their interaction with bacteria, PAM-Tang cell monolayers were infected at 37 °C with *S. suis* 700794 or *S. suis* W7119 stained with 3, 3-dioctadecyloxacarbocyanine perchlorate (DiOC18 (3), Beyotime, China). To visualize nuclei and cell death, the cells were treated with NucBlue™ Live Cell Stain (Hoechst 33342; Thermofisher, Beijing, China) and propidium iodide (PI; BD Biosciences, Franklin Lakes, NJ, USA), respectively.

### 2.9. Western Blotting

PAM-Tang cells were cultured in 12-well plates at a density of 1 × 10^6^ cells/well in RPMI 1640 supplemented with 10% FBS. Thirty minutes prior to infection, the medium was replaced with RPMI 1640 with 3% FBS. Cells were then infected with *S. suis* 700794 or *S. suis* W7119 (MOI = 1) for 0, 9, or 16 h, and subsequently incubated on ice with cell lysis buffer containing PMSF (Solarbio, Beijing, China) and EDTA-free protease inhibitor cocktail (Roche, Merck KGaA, Mannheim, Germany) for 5 min. Protein concentrations were determined using Bicinchoninic Acid Protein Assay kit (Beyotime, Beijing, China). Equal amounts of protein were separated on a 12% SDS-PAGE gel and transferred to polyvinylidene fluoride (PVDF) membranes (MerckMillipore, Darmstadt, Germany) for 1 h. After blocking with 5% dry milk dissolved in PBS at 4 °C overnight, membranes were incubated for 1 h at room temperature with different antibodies: anti-caspase-3, anti-apoptosis-inducing factor (AIF) (1:1000, ABclonal), and anti-β-actin (1:200,000; ABclonal). After washing, the membranes were incubated with the appropriate secondary antibodies for 1 h, and visualized on the near-infrared fluorescence scanning imaging system (Odyssey CLx, Licor, Lincoln, NE, USA) to detect the target bands.

### 2.10. Statistical Analysis

Numerical data are expressed as the mean ± standard deviation, and were analyzed using GraphPad Prism software (version 8.0.1; GraphPad Software Inc.). Significant differences among groups were measured with ordinary one-way ANOVA multiple-comparison test. The comparison of multiple sample sets vs. control was performed with two-way ANOVA and Tukey’s multiple-comparison test. Differences between groups were assessed using one-way ANOVA and the multiple comparisons. A *p* value less than 0.05 was set as the threshold for significance.

## 3. Results

### 3.1. Avirulent S. suis Presented Higher Adhesion to PAM-Tang Cells

**Figure 1:**
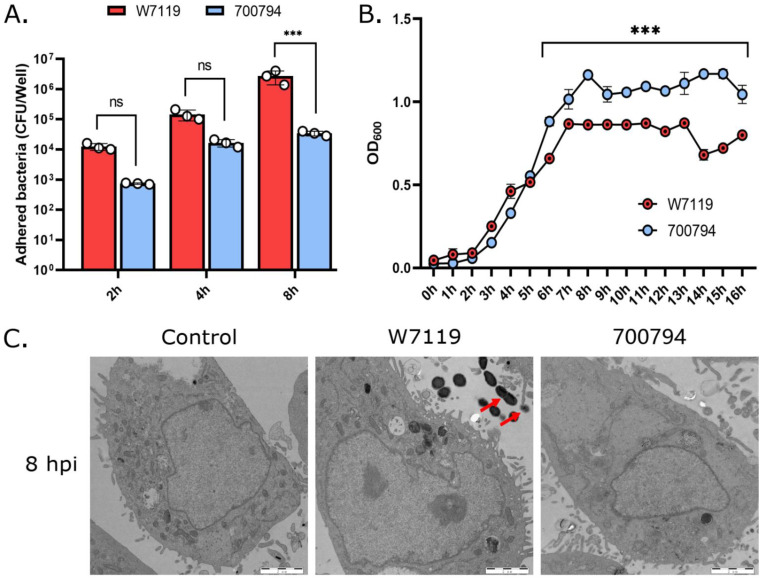
Adhesion to PAM-Tang cells by virulent and avirulent *S. suis*. (**A**) PAM-Tang cells were infected with *S. suis* vir-700794 or *S. suis* avir-W7119 (MOI = 1) for 2, 4, or 8 h. The white circles represent three replicate wells. (**B**) The growth of the two strains was tested in THB medium, with OD_600_ values measured every hour. (**C**) Electron micrographs of PAM-Tang cells at 8 h post-infection (hpi) with virulent or avirulent *S. suis*. Ultrathin section, scale bar = 2 μm. Red arrows showed *S. suis*. All treatments were carried out in triplicate and experiments were repeated at least three times, results are expressed as means ± S.D, two-way ANOVA used to analyze the data; ns: not significant; ***: *p* < 0.001.

The kinetics of adhesion were compared for *S. suis* isolates of different virulence. Adhesion of *S. suis* to PAM-Tang cells was time-dependent in both virulent and avirulent strains (Figure 1A). By 8 hpi, the avir-W7119 exhibited significantly more adhesion to PAM-Tang cells than vir-700794. In order to discard that the difference in adhesion was based on the rate of bacterial reproduction, growth curves of virulent and avirulent *S. suis* in THB medium were compared. The growth rate of vir-700794 was actually higher than that of avir-W7119 after 6–8 hpi (Figure 1B). In addition, W7119 does not show bacteria adherent to cells, but to the spaces between cells by TEM (Figure 1C), which is consistent with a hydrophobic bacterium. These results collectively indicate that more adhesion to PAM-Tang cells seems to be associated with less capsule in the avirulent *S. suis* strain.

### 3.2. S. suis Virulent Strain Has Higher Cytotoxic to PAM-Tang Cells

**Figure 2:**
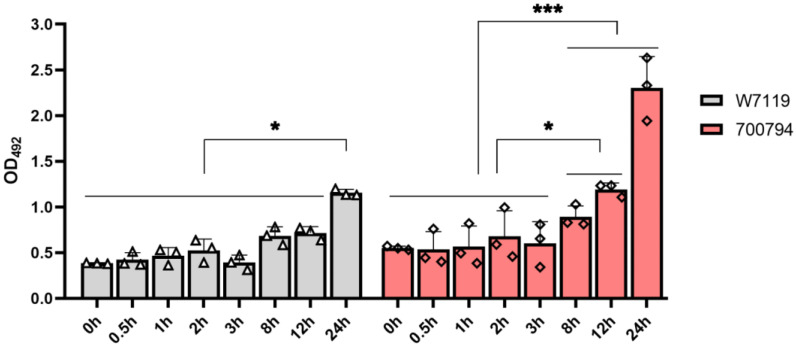
Cytotoxicity induced by *S. suis* infection. PAM-Tang cells were infected with *S. suis* vir-700794 or *S. suis* avir-W7119 at MOI = 1 and incubated for 0.5, 1, 2, 3, 8, 12, or 24 h, negative control expressed as 0 h. The triangles represent *S. suis* W7119 infection group (*n* = 3), diamonds represent *S. suis* 700794 infection group (*n* = 3). Culture supernatants were measured for their LDH activity and expressed as optical density (OD_492_). Each number represents the mean ± SD generated from three cell supernatants at each time point, and experiments were repeated three times. significance was determined using two-way ANOVA and Tukey’s multiple-comparison test; ns: not significant; *: *p* < 0.05; ***: *p* < 0.001.

In order to determine if *S. suis* could be cytotoxic to PAM-Tang cells, LDH release was measured in cell culture supernatants at different times post-infection. Both *S. suis* strains 700794 and W7119 were cytotoxic to PAM-Tang cells, with LDH activity increasing throughout the culture time (Figure 2). However, it should be noted that the levels of cytotoxicity of both strains were very low during early infection. By 12 hpi, the level of cytotoxicity of vir-700794 was significantly higher than that of avir-W7119 in PAM-Tang cells. These results suggest that induction of cytotoxic damage to PAM-Tang cells was positively correlated with *S. suis* virulence.

### 3.3. S. suis Induces PAM-Tang Polarization towards Pro-Inflammatory M1 Macrophages

**Figure 3:**
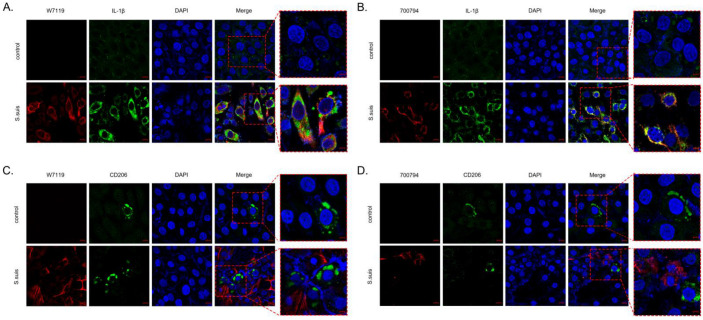
Confocal laser scanning microscopy images of *S. suis* inducing PAM-Tang polarization. PAM-Tang cells were infected by *S. Suis* vir-700794 or *S. suis* avir-W7119 (MOI = 1) and stained with anti-*S. suis* antibody (red), M1 macrophage marker IL-1β antibody (green) in (**A**,**B**), or M2 macrophage marker CD206 antibody (green) in (**C**,**D**). Nuclei were stained by DAPI (blue).

Macrophages are major regulators of inflammation, and can polarize into different phenotypes based on perceived stimuli [22,23]. To investigate the effect of *S. suis* on macrophage polarization during *S. suis* infection, PAM-Tang cells were infected with strain 700794 or W7119. IL-1β expression is a marker of pro-inflammatory M1 macrophages, and it increased significantly by 8 hpi in both *S. suis* strains (Figure 3A,B). Meanwhile, expression of CD206 (marker of anti-inflammatory M2 macrophages) was increased only slightly (Figure 3C,D). According to these results, PAM-Tang M1 polarization does not seem to be an exclusive hallmark of virulent *S. suis*.

### 3.4. S. suis Induces PAM-Tang Cells to Secrete Pro-Inflammatory Cytokines

**Figure 4:**
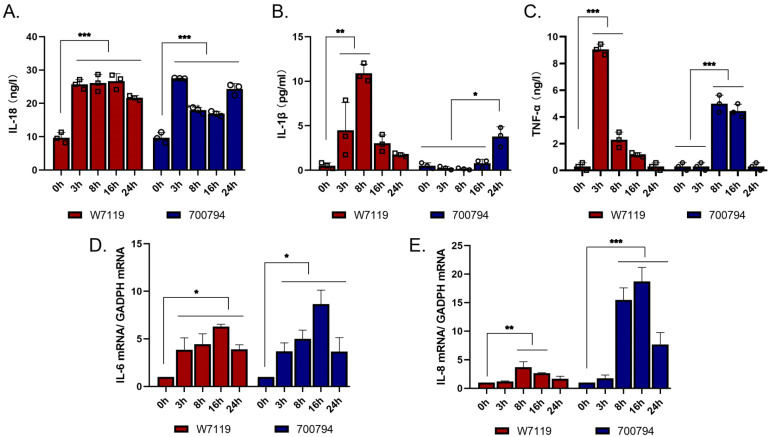
*S. suis* induces expression of inflammatory mediators in PAM-Tang cells. PAM-Tang cells were infected with *S. suis* vir-700794 or *S. suis* avir-W7119 at MOI = 1 for 3, 8, 16, or 24 h, negative control expressed as 0 h. After infection, ELISA kits were used to detect culture supernatants levels of IL-18 (**A**), IL-1β (**B**), and TNF-α (**C**). The squares represent *S. suis* W7119 infection group (*n* = 3), circles represent *S. suis* 700794 infection group (*n* = 3). Quantitative RT-PCR assay was used to detect transcriptome levels of IL-6 (**D**) and IL-8 (**E**) in cells after infection. All treatments were carried out in three wells, and experiments were repeated three times. The data used for statistical analysis represent the mean ± SD of cytokines secreted into the cell supernatant at each time point, significance was determined using two-way ANOVA and Tukey’s multiple-comparison test; *: *p* < 0.05; **: *p* < 0.01; ***: *p* < 0.001.

To determine if PAM-Tang cells exposed to *S. suis* have the ability to secrete pro-inflammatory cytokines, we used ELISA kits to detect the pro-inflammatory cytokines IL-18, IL-1β, and TNF-α secreted by PAM-Tang cells at different times post-infection. Both virulent and avirulent *S. suis* strains induced the secretion of pro-inflammatory cytokines. IL-18 secretion was increased significantly by both avirulent and virulent strains, and remained high from 3 to 24 hpi (Figure 4A). IL-1β secretion was significantly higher in both *S. suis* strains, but there were differences in the timing of the avirulent (increased at 3 hpi, peak of 3.78 pg/mL at 8 hpi) vs. the virulent (increased at 24 hpi, 10.89 pg/mL) (Figure 4B). Similarly, TNF-α increased significantly in the avirulent strain earlier (3 and 8 hpi) than the virulent strain (8 and 16 hpi) (Figure 4C). Overall, both strains induced secretion of pro-inflammatory cytokines in PAM-Tang cells.

In order to strengthen the results of pro-inflammatory cytokines, we used quantitative RT-PCR assay to test transcriptome levels of IL-6 and IL-8 in cells after infection. The data of IL-6 and IL-8 were expressed as IL-6 or IL-8 mRNA/GADPH mRNA. As shown in Figure 4D,E, transcriptome levels of IL-6 and IL-8 were significantly higher in both *S. suis* strains, but there were differences in the time at which they start to rise and last; IL-6 starts to rise earlier and last longer than IL-8.

### 3.5. PAM-Tang Phagocytosis of S. suis

**Figure 5:**
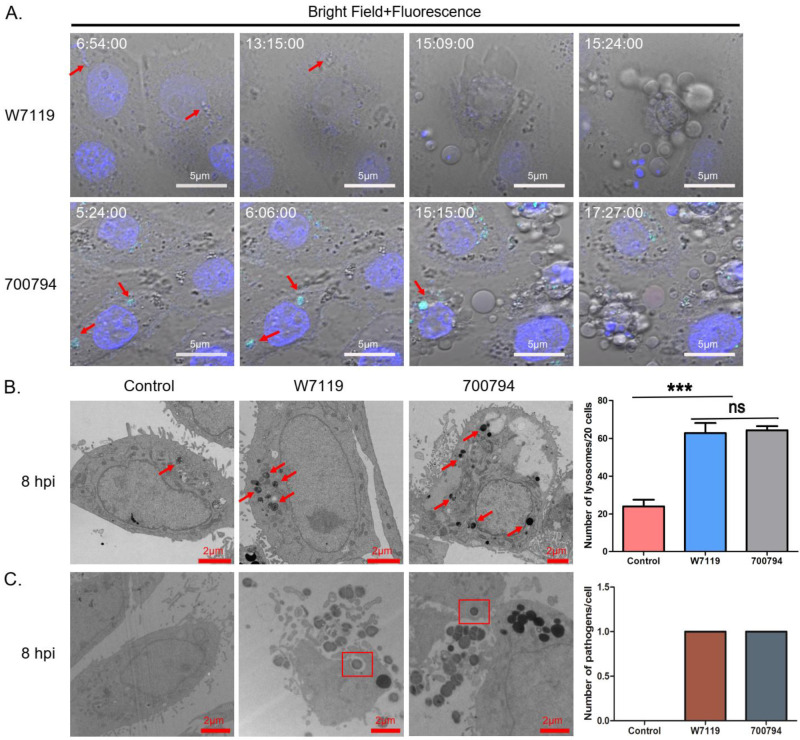
Tracking the phagocytosis of PAM-Tang cells. (**A**) Live-cell imaging of phagocytosis by PAM-Tang cells after infection with fluorescent-labeled (green) *S. suis* vir-700794 or unlabeled *S. suis* avir-W7119 (MOI = 1). The red arrows indicate *S. suis* cells, and numbers at upper left indicate infection time (h:min:sec); cell nuclei (blue) were stained with Hoechst 33342. (**B**) Electron micrographs of macrophages showing a significant increase in number of lysosomes (red arrows) in cells at 8 h post-infection (hpi) by *S. suis.* (**C**) Electron micrographs of phagocytic vacuole (red rectangle) in PAM-Tang at 8 h post-infection (hpi) by *S. suis.* Experiments were repeated three times, results are expressed as means ± S.D, significance was determined using one-way ANOVA multiple-comparison test; ***: *p* < 0.001; ns: not significant.

To determine whether PAM-Tang cells are capable of phagocytosis, we infected them with *S. suis* strain 700794 or strain W7119 and monitored them by live-cell imaging. By 7 hpi, avir-W7119 bacteria appeared into view, and after several hours, PAM-Tang cells began to phagocytose and eliminate them (Figure 5A; also see Supplementary Video S1). During this process, PAM-Tang cells produced a large number of vesicles, releasing them continuously. Similarly, PAM-Tang cells infected with vir-700794 constantly devoured the fluorescent-labeled bacteria, while producing a large number of vesicles. The number of lysosomes may increase after bacteria are phagocytosed in cells [24], so we counted the lysosomes in 20 PAM-Tang cells by TEM. The result showed that the number of lysosomes in the infected PAM-Tang cells was significantly increased compared with control cells (Figure 5B). In addition, *S. suis* was observed within phagocytic vacuoles in PAM-Tang cells (Figure 5C). We found 10 cells with bacteria in 10 fields under TEM, and the number of bacteria was 1 per cell (Figure 5C). These results proved that the PAM-Tang cell line has the capability to phagocytose *S. suis*.

### 3.6. S. suis Induces Apoptosis in PAM-Tang Cells

**Figure 6:**
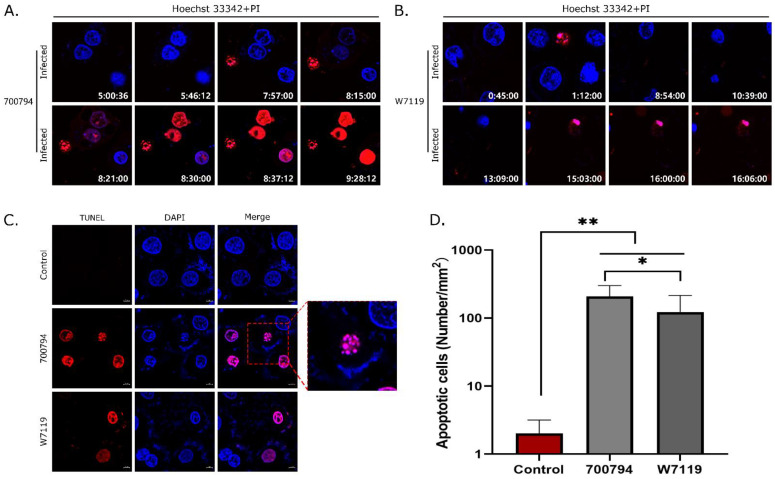
Identification of cell death mode in infected PAM-Tang cells. PAM-Tang cells were infected with (**A**) *S. suis* vir-700794 or (**B**) *S. suis* avir-W7119, and cell death was observed with propidium iodide (PI; red). Numbers at lower right indicate infection time (h:min:sec); viable nuclei were stained with Hoechst 33342. (**C**) Confocal laser scanning microscopy images of apoptosis (red) in PAM-Tang cells infected with *S. suis* (MOI = 1) for 8 h by TUNEL assay; the enlarged red rectangle shows an apoptotic body. Nuclei of the cells were stained with DAPI (blue). (**D**) Apoptotic cells in PAM-Tang infected with *S. suis* (MOI = 1) for 8 h were counted and analyzed using one-way ANOVA multiple-comparison test; *: *p* < 0.05; **: *p* < 0.01. All treatments were carried out in triplicate and experiments were repeated three times.

We used live-cell imaging to investigate apoptosis induced in PAM-Tang cells by *S. suis*. PI is a nucleic acid dye, which can penetrate the cell membrane and stain the nucleus red in the late stage of apoptosis. At about 8 hpi with vir-700794, we observed a few dead cells (Figure 6A) which increased in number by around 10 hpi (Supplementary Video S2). Similarly, cells infected with avir-W7119 began to die, but a bit later, around 15 hpi (Figure 6B). Subsequently, a TUNEL assay detected apoptotic signals colocalizing with cell nuclei, and apoptotic bodies consisting of membrane-bound fragments with condensed cytoplasm and nuclei (Figure 6C). The number of apoptotic cells induced by vir-700794 was significantly higher (*p* < 0.05) than that induced by avir-W7119 (Figure 6D).

### 3.7. S. suis Induces Caspase- and p53-Dependent Apoptosis in PAM Cells

**Figure 7:**
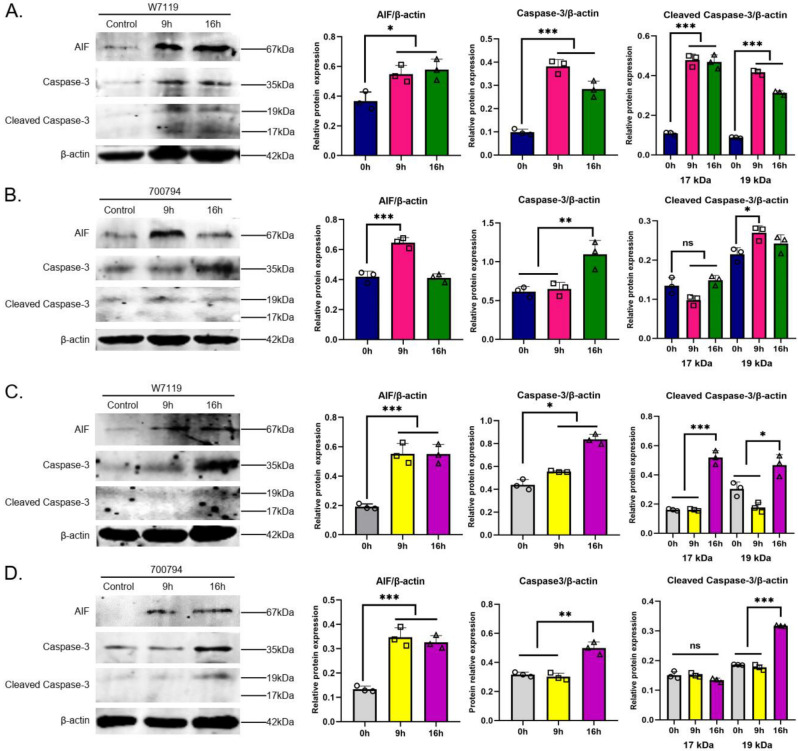
*S. suis* induces caspase- and p53-dependent apoptosis in PAM cells. PAM-Tang cells were infected with (**A**) *S. suis* avir-W7119 or (**B**) *S. suis* vir-700794, and primary PAMs were infected with (**C**) *S. suis* avir-W7119 or (**D**) *S. suis* vir-700794 at an MOI = 1 for 0, 9, and 16 h. The cell lysates were collected, levels of proteins related to apoptotic functional indexes: caspase-3, cleaved-caspase-3, and AIF were determined by Western blot and analyzed digitally, and the optical density ratio was calculated. Three identical graphics represent three independent wells in a single experiment, and experiments were repeated three times; the results are expressed as means ± S.D, significance was determined using one-way ANOVA multiple-comparison test; ns: not significant; *: *p* < 0.05; **: *p* < 0.01; ***: *p* < 0.001.

Next, we explored which signaling pathways participate in *S. suis*-induced PAMs apoptosis. Mitochondrial AIF is a proapoptotic factor in the p53 signaling pathway that is translocated from mitochondria to the nucleus, causing chromatin condensation and nuclear fragmentation [25]. Both virulent and avirulent *S. suis* strains induced a significant increase in AIF protein in PAM-Tang or primary PAM cells at 9 hpi and 16 hpi (Figure 7A–D), and only strain 700794 did not induce increased AIF in PAM-Tang cells at 16 hpi.

Caspase-3, functioning as the most important executioner caspase, cleaved various substrates downstream and ultimately caused the biochemical and morphological changes seen in apoptotic cells [26]. Activation of caspase-3 requires proteolysis of its inactive proenzyme form into activated p17 and p12 fragments. Expression of caspase-3 and cleaved caspase-3 increased significantly after infection with either virulent or avirulent *S. suis* (Figure 7A–D). Taken together, these results showed that *S. suis*-induced apoptosis is dependent on the caspase and AIF/p53 signaling pathways.

## 4. Discussion

Since alveolar macrophages serve as the first line of defense against bacterial infection in the lung, it is important to investigate their interaction with pathogens such as *S. suis*. The macrophage cell line J774 is derived from BALB/c mice, and has been used to study the effects of *S. suis* infection [12]. However, since *S. suis* is a swine pathogen, it is often questioned whether mouse-origin cells can truly reflect the complex biological relationship between the cells [27]. Primary alveolar macrophages have been proliferated indefinitely by transfecting the human telomerase reverse transcriptase (hTERT) cDNA [28]. Our lab has also used hTERT to immortalize primary PAMs (data not shown), but we could not passage them well. The continuous PAM line 3D4 were established following transfection of primary PAMs with plasmid pSV3neo, carrying the SV40 large T antigen gene, but it exhibited nonessential amino acid-dependent growth [29]. In contrast, PAM-Tang cells are immortalized by integration of a lentivirus carrying the large T antigen SV40 gene into the genome. This allows constitutive expression of the SV40 protein, and can be passaged well without the need for nonessential amino acids in the medium [16]. Thus, the PAM-Tang cell line is cultured in a normal medium, which is convenient and can reduce the cost of the experiment. Though PAM-Tang cells with high CD163 abundances have been used in PRRSV research for several years, this is the first report of *S. suis* interaction with PAM-Tang cells with low CD163 abundance (approximately 20%).

Our results are consistent with several reports that have shown that bacteria can adhere to macrophages [12]. It has been previously reported that the low- or non-virulent *H. parasuis* strain adhered to PAMs at significantly higher levels than virulent strains [11,30]. Similarly, in the present study, avir-W7119 adhered more strongly to PAM-Tang cells than vir-700794. The strain W7119 has less capsule than the strain 700794 (data not shown), which is likely to render W7119 more hydrophobic and more likely to adhere to the surface of PAM-Tang cells. The electron microscope image of W7119 does not show adherence to cells, but to the spaces between cells, which is consistent with a hydrophobic bacterium. Since the ability of microorganisms to bind to the cell surface contributes to phagocytosis [12], the avirulent strain was phagocytosed more easily by macrophages. As a result, it is possible that avirulent strains have less impact on the host than the virulent strain. Interestingly, the PAM-Tang macrophage cell line was shown to phagocytose bacteria in our study, by both live-cell imaging and TEM. Therefore, PAM-Tang cells may be used to study interactions with *S. suis* in vitro as a cell model.

*S. suis* expresses multiple confirmed and putative virulence factors [31,32,33], including glutamate dehydrogenase (gdh), muramidase-released protein (mrp), suilysin protein (sly), and extracellular protein factor (epf). The virulence phenotype of strain W7119 is *gdh*^+^/*mrp*^−^/*sly*^−^/*epf*^−^, and the virulence phenotype of strain 700794 is *gdh*^+^/*mrp*^+^/*sly*^+^/*epf*^+^ (data not shown). As one of the most important virulence factors of *S. suis*, sly has been proven to be cytotoxic to macrophages in many studies [34,35]. We showed vir-700794 to be relatively more cytotoxic than avir-W7119 at the late stage of infection, and we suspect that this may be due to the existence of sly. Segura et al. confirmed that *S. suis* has a toxic effect on macrophages with increased incubation time [12,27], which is consistent with our results. In addition, the release of LDH is used to measure cytotoxicity; LDH also can be released by cells undergoing pyroptosis in response to inflammasome activation [36]. In this study, we test the levels of IL-18 in cellular supernatant, which is a downstream product of inflammasome activation in PAMs. As a result, both strains induced an IL-18 increase from 3 hpi to 24 hpi, which indicates that PAMs-Tang may undergo pyroptosis after *S. suis* infection. This will be confirmed in future studies. Accordingly, the LDH increase from 8 hpi to 24 hpi may be contributed by pyroptosis. The nuclear factor-kappa B (NF-κB) signaling pathway is a prototypical pro-inflammatory pathway and is induced by toll-like receptor (TLR) [37], and it’s activation promotes pro-IL-1β to mature IL-1β. Our results show that avir-W7119 induces IL-1β increase from 3 hpi to 8 hpi, while vir-700794 induces IL-1β increase mainly at 24 hpi, which indicates that avir-W7119 may be more likely to bind to TLR and activate the NF-κB pathway earlier than vir-700794, and then facilitate elimination.

*S. suis* infection is often accompanied by inflammation [38], and as the frontline cells of innate immunity, macrophages play a crucial role in this entire process [23,39]. Macrophages exhibit different phenotypes and can shift between M1-type and M2-type to repair inflammation and maintain homeostasis [40,41]. Our work is the first to show an induction of M1-type macrophage polarization in vitro by *S. suis*, which induces the secretion of pro-inflammatory cytokines. The type of polarization was consistent with macrophages in the spleens of mice induced by *S. suis* [42]. The expression of proinflammatory cytokines (TNF-a, IL-8, IL-1-β, IL-6, and MIP-1-β) was increased in macrophages, 3D4 incubated with the *S. suis* strains by qPCR analysis [13]. Similarly, in our study, an increase in proinflammatory cytokine production (IL-6, IL-8, IL-18, IL-1β, and TNF-α) was seen in PAM-Tang cells after *S. suis* incubation by quantitative RT-PCR and quantifying cytokine assay. Altogether, these results indicate that proinflammatory responses are dominant after *S. suis* infection.

Live-cell imaging with PI dye combined with TUNEL detection showed a significant induction of apoptosis by *S. suis* infection. Apoptosis is a tightly controlled, multi-step cell death mechanism, and may occur via death mitochondrial (intrinsic) or receptor-dependent (extrinsic) pathways. The p53 (intrinsic) pathway is mainly mediated by mitochondria-associated AIF, and the caspase (extrinsic) pathway is mainly mediated by caspase-3 [28]. Furthermore, our results of Western blot assays showed that both virulent and avirulent *S. suis* strains induced caspase- and p53- dependent apoptosis in PAM-Tang and primary PAM cells, which were consistent with the signaling pathway of thymocyte apoptosis in the thymus of mice induced by *S. suis* [18]. These findings clearly confirm that macrophages undergo apoptosis through both the extrinsic and the intrinsic pathways in *S. suis* infection. The consistency of the *S. suis* induced apoptosis signaling pathway between PAM-Tang and primary PAMs further indicates that PAM-Tang cells could instead be primary PAMs. Overall, the PAM-Tang cell line may be used in the study of interactions with *S. suis* in vitro as a suitable cell model.

## 5. Conclusions

In conclusion, we identified, for the first time, interactions between PAM-Tang cells and *S. suis* strains with different virulence. Both strains induced the phagocytosis and M1-type polarization of PAM-Tang cells, as well as the secretion of pro-inflammatory cytokines. Moreover, it is the first report of virulent and avirulent *S. suis* infection inducing p53- and caspase-dependent apoptosis in PAMs. In summary, our results highlight PAM-Tang cells as a valuable cell model for the study of *S. suis* infection in vitro.

## Data Availability

Data are available in section “MDPI Research Data Policies” at https://www.mdpi.com/ethics.

## Supplementary Materials

The following supporting information can be downloaded at: https://www.mdpi.com/article/10.3390/microorganisms11010160/s1, Video S1: PAM-Tang cells phagocytose *S. suis*; Video S2: Dead cells increased in number.
